# When poverty gets into your joints: exploring socioeconomic, environmental, and gendered determinants of rheumatoid arthritis in district Dir lower, Pakistan

**DOI:** 10.3389/fpubh.2025.1706246

**Published:** 2026-01-05

**Authors:** Younas Khan, Umar Daraz, Rula Odeh Alsawalqa, Maissa N. Alrawashdeh, Ann Mousa Alnajdawi

**Affiliations:** 1 Department of Management Science and Engineering, School of Business, East China University of Science and Technology, Shanghai, China; 2 Department of Sociology, University of Malakand, Chakdara, Pakistan; 3 Department of Sociology, The University of Jordan, Amman, Jordan; 4 Department of Social Work, The University of Jordan, Amman, Jordan

**Keywords:** rheumatoid arthritis, health disparities, social determinants of health, patient-reported outcomes, rural health, Pakistan, gender and health, low- and middle-income countries

## Abstract

**Introduction:**

Rheumatoid Arthritis (RA) imposes a significant disease burden in low-and middle-income countries (LMICs), yet evidence on its socio-structural factors in underserved populations remains scarce. This study investigates the key environmental, gendered, and structural factors influencing patient-reported experiences of RA among women in rural Pakistan, where access to consistent rheumatology care is extremely limited (<5%).

**Methods:**

An epidemiological cross-sectional study was conducted with 300 women with RA in rural Pakistan. Data were collected via structured interviews assessing self-reported pain, perceived mobility limitation, psychological stress, and treatment adherence. Statistical analyses employed ordinal logistic regression and Structural Equation Modeling (SEM) to identify and model the determinants and pathways influencing patient-reported outcomes.

**Results:**

Analysis revealed three primary determinants: (1) Climate-linked disease burden: Colder winter temperatures were significantly associated with exacerbated self-reported pain (Z = 12.24, *p* < 0.001, *r* = 0.52). (2) Gendered care barriers: Spousal unemployment doubled the risk of treatment dropout (U = 5690.5, *p* < 0.001). (3) Structural marginalization: Residing >50 km from a specialist care center was associated with 3.2 times higher odds of reporting severe perceived mobility limitations (χ^2^ = 28.49, *p* < 0.001). SEM confirmed significant interconnected pathways, demonstrating how income loss and psychological stress mediate adverse patient-reported outcomes (CFI = 0.94, RMSEA = 0.042).

**Discussion:**

As one of the first studies to quantify seasonal and gender-based disparities in RA in South Asia, our findings underscore the critical role of socio-structural factors in shaping disease experience in resource-poor settings. The results directly inform the WHO Global RA Action Plan for LMICs. We propose three evidence-based interventions for underserved regions: mobile rheumatology units to bridge geographical gaps, climate-adaptive pain management protocols, and community-led psychosocial support programs to address gendered and economic vulnerabilities.

## 1 Introduction

Rheumatoid arthritis (RA) is a chronic, disabling autoimmune condition characterized by persistent joint inflammation, progressive immobility, and systemic complications that impair quality of life (1). While RA has long been interpreted primarily through a biomedical lens, a growing body of global evidence shows that its onset, severity, and long-term outcomes are profoundly influenced by the social determinants of health (2, 3). Rather than existing in a vacuum, RA unfolds within complex socio-ecological environments shaped by economic deprivation, gendered labor roles, cultural expectations, healthcare inequities, and climate-related stressors (4). Collectively, these conditions can exacerbate inflammation, delay diagnosis, and heighten disability, particularly for marginalized populations.

A consistent pattern across international research affirms that socioeconomic status (SES) significantly determines RA risks, disease progression, and treatment continuity. Individuals with limited income, unstable employment, and low education levels are far more likely to experience severe joint damage, delayed access to rheumatology services, and treatment non-adherence (5). A systematic review further affirmed that socioeconomic deprivation is tied to increased disease activity and poorer long-term outcomes (6). Beyond socioeconomic inequality, environmental stressors such as cold temperatures, damp conditions, and seasonal climate variability have also been linked to increased joint pain, stiffness, immobility, and higher dropout rates from medication (7, 8). Patients living in cold, poorly heated environments or regions with harsh winters face additional barriers to maintaining mobility and attending clinical appointments (9).

Parallel to these ecological and socioeconomic factors, access to specialized rheumatology care represents another critical dimension of RA inequality. In many low- and middle-income countries (LMICs), structural barriers—long travel distances, high out-of-pocket costs, insufficient specialists, and fragmented health systems—restrict timely treatment (10, 11). These limitations drive many patients, especially in rural settings, to rely on informal or traditional therapies whose effectiveness is shaped by poverty, cultural norms, and environmental context (12–14). Gender norms compound these barriers: women globally face both biological susceptibility to autoimmune diseases and disproportionate responsibility for unpaid household labor. Even with severe joint pain, women often continue physically demanding caregiving duties, which accelerates disease progression and delays recovery (15–17).

### 1.1 Regional context: RA in rural Pakistan

In Pakistan, the social determinants that worsen RA are magnified by deep structural inequalities, limited healthcare coverage, entrenched gender disparities (18), and recurring climate shocks (19). Over 70% of healthcare expenditures in Pakistan are out-of-pocket, pushing millions into poverty when attempting to manage chronic illnesses such as RA (20, 21). A cross-sectional study in Lahore found that household income and education level were the most significant predictors of delayed RA diagnosis and reduced treatment adherence (22). Harsh winters in the northern and north-western regions—including Khyber Pakhtunkhwa—further intensify RA symptoms, especially in homes lacking insulation, heating, or reliable electricity (23, 24). A recent study by Zulfiqar and Prasad (25) reported that 58% of RA patients in rural Punjab had never visited a rheumatologist due to cost, distance, or social stigma.

Furthermore, cultural expectations place disproportionate burdens on women. In many rural households, women are expected to continue domestic labor—cooking, cleaning, washing, childcare, carrying firewood—even when experiencing debilitating RA symptoms (26). Thus, household income, employment status, education level, distance to healthcare facilities, domestic labor demands, and climate extremes constitute critical yet underexplored factors within the lived experiences of RA sufferers in low-resource contexts (27).

### 1.2 Rationale for the study

The motivation for this study stems from recurring clinical observations that patients in District Dir Lower, Khyber Pakhtunkhwa, experience heightened RA-related disability during the winter months. These patterns cannot be explained through biological mechanisms alone; they are deeply connected to structural realities, including unheated homes, chronic poverty, limited medication access, long travel distances to the nearest specialist, and rigid patriarchal caregiving norms. In this region, rheumatology services are severely limited: a single visiting rheumatologist serves multiple districts—including Chitral, Bajaur, and Upper Dir—resulting in wait times of up to 2 months for very brief consultations. Patients often travel long distances along mountainous terrain during severe cold spells, which exacerbates pain and restricts access to timely care.

Poverty intensifies these vulnerabilities: over 60% of households rely on informal work or subsistence agriculture (28). Women, in particular, are doubly marginalized—expected to maintain physically demanding domestic labor regardless of health status and rarely prioritized for medical treatment. Interviews and clinical narratives reveal that many women skip treatment due to household responsibilities, financial constraints, and climate-induced immobility. These intersecting disadvantages signal a form of structural violence embedded within everyday life.

Despite the urgency of these issues, existing RA research in Pakistan remains overwhelmingly biomedical, with minimal attention to gendered, ecological, and socioeconomic realities. Although international studies have affirmed the impact of cold climates, socioeconomic deprivation, and healthcare inaccessibility on RA outcomes, there is a critical knowledge gap regarding the combined influence of these factors in rural Pakistan.

Guided by an eco-social theoretical framework (29), this study seeks to fill that gap by examining how economic hardship, environmental exposure, and patriarchal caregiving norms collectively shape disease progression, disability, and coping strategies among women with RA in Dir Lower. Through this lens, RA is reframed not merely as a biological condition but as a socially patterned and structurally produced health inequality.

## 2 Research gaps and objectives

### 2.1 Identified research gap

Despite global and national recognition of RA as a complex chronic condition influenced by social and environmental factors, there is no quantitative study in Pakistan—let alone in district Lower Dir—that integrates the dimensions of poverty, climate sensitivity, healthcare barriers, and gendered labor into a unified empirical model. Previous studies tend to isolate one or two factors, neglecting how these determinants interact systematically to worsen RA conditions. Moreover, while some qualitative research touches on rural health inequalities, there remains a complete absence of large-scale, statistically driven research exploring the unique intersection of seasonal pain, spousal unemployment, treatment discontinuity, and female labor burden from the patients’ perspective. This study seeks to fill this gap by quantitively examining these interconnected socio-structural determinants and their relationship to key patient-reported outcomes, such as pain intensity, functional mobility, and treatment adherence, which are critical to understanding the daily burden of RA in resource-poor settings.

### 2.2 Objectives of the study

1. To examine the relationship between household income, employment status, and the severity of RA symptoms among women in District Dir Lower.
2. To assess how seasonal climatic conditions, particularly winter, affect RA pain intensity, mobility, and treatment adherence.
3. To investigate the barriers to accessing rheumatology care, including distance, transport, cost, and stigma.
4. To analyze the impact of gendered domestic labor and spousal unemployment on psychological stress and disease management.
5. To explore the coping strategies employed by female RA patients in winter and evaluate their effectiveness.

## 3 Theoretical framework

### 3.1 Eco-social theory foundation

This study is anchored in Nancy Krieger’s Eco-Social Theory of Disease Distribution, which posits that health disparities arise from the complex interplay between social, economic, political, and environmental contexts (29, 30). The theory emphasizes the concept of “embodiment,” whereby adverse living conditions—such as poverty, gender inequity, and environmental stressors—are biologically absorbed over time, leading to poor health outcomes (31). Central to the eco-social framework is the understanding that structural inequities (e.g., unemployment, limited healthcare access, and social exclusion) shape individual vulnerability to disease, both directly and through cumulative exposures over the life course (32). This holistic perspective allows for analyzing how macro-level determinants manifest at the individual level to worsen chronic illnesses like RA.

### 3.2 Linking eco-social theory to the study objectives

Eco-Social Theory is particularly relevant to the current study as it accommodates the multidimensional nature of RA determinants. Objective 1, which examines the role of household income and employment status, directly reflects the eco-social focus on socioeconomic stratification as a driver of disease burden. Objective 2, investigating how seasonal climatic conditions exacerbate pain and disability, aligns with the theory’s emphasis on environmental exposures and their embodiment in physical health. Objective 3, which explores barriers to healthcare access, draws on the theory’s assertion that structural barriers—such as geographic isolation and unaffordable medical care—shape inequitable disease outcomes. Objective 4 addresses the gendered dimensions of domestic labor and spousal unemployment, linking closely to the eco-social perspective on power hierarchies and how gender-based inequities affect health trajectories. Finally, Objective 5 examines coping strategies, which the eco-social framework recognizes as adaptive responses shaped by social and environmental constraints. By statistically modeling these interconnected factors, this study applies the eco-social lens to disentangle how poverty, climate vulnerability, healthcare inaccessibility, and gender roles jointly influence RA severity in District Dir Lower.

### 3.3 Unique contributions and theoretical advancement

While Eco-Social Theory has been extensively applied to understand health inequalities globally, few empirical studies have operationalized it in the context of RA in low-resource, climate-vulnerable settings like rural Pakistan. Most prior research informed by the theory has focused on broader public health conditions (e.g., infectious diseases or malnutrition), rather than chronic autoimmune conditions with gendered and environmental dimensions. This study contributes uniquely by quantitatively testing the eco-social framework in relation to seasonal pain fluctuations, gendered caregiving burdens, and healthcare discontinuities—factors that have rarely been examined together. Moreover, by integrating indicators specific to Khyber Pakhtunkhwa’s sociocultural and geographic context (e.g., male breadwinner unemployment, long distances to rheumatologists, winter coping strategies), this research extends the eco-social theory’s explanatory power to rural South Asian populations. In doing so, the study addresses a theoretical gap by demonstrating how eco-social determinants interact cumulatively in under-researched populations, thereby strengthening the framework’s global applicability and informing more targeted interventions for chronic disease management.

## 4 Methodology

### 4.1 Research design

This study employed a cross-sectional quantitative research design to statistically examine the relationships between socioeconomic status, seasonal climatic conditions, healthcare access, gendered responsibilities, and coping strategies, and the severity and management of RA (33).

### 4.2 Study area

The research was conducted in District Dir Lower, a mountainous and underdeveloped region in Khyber Pakhtunkhwa (KP), Pakistan. The area is characterized by harsh winters, poor infrastructure, and limited access to specialized healthcare. Rheumatology services are provided by a single visiting rheumatologist at the District Headquarters Hospital (DHQ) in Timergara once a week, serving patients from Dir Lower, Upper Dir, Bajaur, and Chitral, with average wait times exceeding 2 months.

### 4.3 Participants

#### 4.3.1 Inclusion and exclusion criteria

The study primarily focused on female RA patients to address the core research objectives related to gendered determinants. The inclusion criteria were adults (aged 20+) with a confirmed or suspected RA diagnosis, residing in Dir Lower. Although the recruitment strategy prioritized women, 24 male patients who met the criteria and presented at the clinic during the data collection period were also enrolled, resulting in a final sample that was 92% female (*n* = 276). This allows for a limited exploration of gender disparities within this specific context.

#### 4.3.2 Sample size and sampling techniques

A total of 300 participants were selected using a purposive sample size is justified by power analysis standards for Structural Equation Modeling (SEM), which recommends 10–15 cases per estimated parameter. The recruitment took place during the weekly rheumatology clinic at DHQ Hospital Timergara.

#### 4.3.3 Participants characteristics

The socioeconomic and demographic profile of the participants is presented in Table 1. The sample was predominantly female (92%), with the largest age group being 35–49 years (39%). Most participants were married (88%), had no formal education (46%), and lived in low-income households (73% earned ≤PKR 30,000/month). The majority lived in semi-permanent or mud housing (73%) and relied on wood/biomass (53%) or had no heating (33%) during the winter season.

**Table 1:** Socioeconomic and demographic profile of participants (*N* = 300).

Variable	Categories	Frequency (n)	Percentage (%)
Gender	Female	276	92.0%
	Male	24	8.0%
Age group (years)	20–34	48	16.0%
	35–49	117	39.0%
	50–64	93	31.0%
	65 and above	42	14.0%
Marital status	Married	264	88.0%
	Widowed	21	7.0%
	Divorced	15	5.0%
Education level	No formal education	138	46.0%
	Primary (up to Grade 5)	69	23.0%
	Secondary (Grade 6–10)	57	19.0%
	Intermediate or higher	36	12.0%
Employment status of primary earner	Employed (formal/informal)	189	63.0%
	Unemployed	111	37.0%
Household monthly income (PKR)	Less than 15,000	96	32.0%
	15,001–30,000	123	41.0%
	30,001–50,000	57	19.0%
	Above 50,000	24	8.0%
Household size	1–4 members	42	14.0%
	5–7 members	183	61.0%
	8 or more members	75	25.0%
Housing type	Mud/Thatched	87	29.0%
	Semi-permanent (brick/mud mix)	132	44.0%
	Permanent (brick/cement)	81	27.0%
Source of heating in winter	None	99	33.0%
	Wood/Biomass	159	53.0%
	Electric/Gas heater	42	14.0%

### 4.4 Measures

#### 4.4.1 Instruments structure and content

A structured, close-ended questionnaire was developed based on the study’s objectives and theoretical framework. It was organized into five sections: (1) socioeconomic and demographic sensitivity, (2) Diseases Burden and Climate Sensitivity (e.g., pain intensity by season, mobility limitations), (3) Barriers to healthcare access (e.g., distance, cost, treatment discontinuation), (4) Psychological and Gendered Stressors (e.g., domestic workload, stress levels), and (5) Coping and Adaptation Strategies (e.g., use of heating, traditional remedies).

#### 4.4.2 Indexation and scoring

The measurement and coding of all variables are detailed in Table 2 through application of Likert scale procedure.

**Table 2:** Indexation and measurement of variables used in the study (*N* = 300).

Variable	Type	Scale of measurement	Coding/range	Linked objective(s)
Household income	Independent	Ordinal/Categorical	<15,000 = 1; 15,000–30,000 = 2; >30,000 = 3	1
RA pain intensity (General)	Dependent	Ordinal	1 = Mild, 5 = Severe	1
RA pain intensity (Seasonal)	Dependent	Ordinal	1 = Mild, 5 = Severe (for both summer & winter)	2
Husband’s employment status	Independent	Categorical (Binary)	Employed = 0; Unemployed = 1	4
Stress level of women	Dependent	Ordinal	1 = Low, 5 = High	4
Distance to rheumatologist	Independent	Ordinal / Categorical	<10 km = 1; 10–50 km = 2; >50 km = 3	3
Treatment discontinuation	Dependent	Categorical (Binary)	Continued = 0; Discontinued = 1	3
Winter mobility limitation	Dependent	Ordinal	1 = No limitation, 4 = Severe limitation	1, 2, 4
Coping strategy used	Independent	Nominal	Indoor heating, Remedies, Inactivity, Outdoor work	5
Perceived effectiveness of coping	Dependent	Categorical (Binary)	Not effective = 0; Effective = 1	5
Winter season (Condition)	Independent	Dichotomous (Dummy Variable)	0 = Summer, 1 = Winter	2
SEM latent constructs	Composite	Latent via multi-item scales	Based on aggregated indicators per path	All Objectives via SEM

### 4.5 Questionnaire development and validation

#### 4.5.1 Translation and cultural adaptation

The questionnaire was developed in English, translated into Pashto, and back-translated to ensure conceptual equivalence and cultural appropriateness.

#### 4.5.2 Pilot study

The instrument was pretested with 20 participants from a nearby tehsil Adenazi (not included in the final sample) to refine item wording and assess clarity.

#### 4.5.3 Validity and reliability testing

Content validity was established through expert review by one rheumatologist and two public health scholars. Construct validity was assessed using exploratory factor analysis for multi-items scales. Thus, internal consistency reliability was confirmed with Cronbach’s alpha, exceeding 0.70 thresholds for all relevant scales.

### 4.6 Procedure

#### 4.6.1 Data collection procedure and personal training

The data were collected over six consecutive weeks during Sunday outpatient sessions. Trained female data collectors, fluent in Pashtu and with public health backgrounds, administered the survey face-to-face to ensure comfort and accuracy, particularly for participants with low literacy.

#### 4.6.2 Data management and quality control

Verbal informed consent was obtained from all participants prior to the interview, in line with ethical guidelines for low-literacy populations. All responses were anonymized to ensure confidentiality.

#### 4.6.3 Data analysis plan

Data for the present study were analyzed using IBM SPSS Statistics version 26. The software facilitated a comprehensive range of statistical procedures appropriate for the research objectives and the nature of the data. Descriptive statistics were first employed to summarize the socioeconomic and demographic characteristics of the sample. Inferential tests were then applied to examine the relationships between variables: the Kruskal–Wallis H test assessed differences in RA pain intensity across income levels; the Wilcoxon Signed-Rank Test compared seasonal variations in pain; the Mann–Whitney U test evaluated the impact of spousal unemployment on women’s stress levels; and the Chi-square tests were used to assess associations between healthcare access barriers and treatment discontinuation, as well as to evaluate the perceived effectiveness of various coping strategies. Additionally, ordinal logistic regression was used to identify predictors of winter mobility limitation, and Structural Equation Modeling (SEM) was conducted to explore the complex interrelations among socioeconomic, climatic, psychological, and disability-related variables. The overall models of the study are given as under:

**Model 1: Kruskal–Wallis Test**: To examine the relationship between household income levels and RA pain intensity.

**Equation**:


$$H=\sum ({\mathrm{Ri}}^{2}/\mathrm{ni})-(N{\left(N+1\right)}^{2})/4.$$

Where,

- *H*: Kruskal–Wallis H statistic
- *Ri*: Sum of ranks for group *i*
- *ni*: Number of observations in group *i*
- *N*: Total number of observations

**Model 2: Wilcoxon Signed-Rank Test**: To compare pain severity between summer and winter seasons.

**Equation**:


Z=(T−0.5−n(n+1)/4)/√[n(n+1)(2n+1)/24].

Where,

- *T*: Smaller of the sum of positive or negative ranks
- *n*: Number of non-zero differences
- *Z*: Standardized test statistic

**Model 3: Mann–Whitney U Test**: To assess whether women with unemployed spouses report higher stress levels.

**Equation**:


$$U=n_{1}n_{2}+(n_{1}(n_{1}+1))/2-R_{1}$$

Where,

- *U*: Mann–Whitney U statistic
- *n₁, n₂*: Sample sizes of the two groups
- *R₁*: Sum of ranks in group 1

**Model 4: Chi-Square Test (Treatment Discontinuation)**: To test the association between distance to rheumatologist and treatment discontinuation.

**Equation**:


$$\chi^{2}=\sum [{\left(O_{i}-E_{i}\right)}^{2}/E_{i}]$$

Where,

- *χ^2^*: Chi-square statistic
- *Oᵢ*: Observed frequency
- *Eᵢ*: Expected frequency

**Model 5: Ordinal Logistic Regression**: To identify predictors of winter mobility limitation in women with RA.

**Equation**:


$$\text{logit}\;(P(Y\leq j))=\alpha_{j}-(\beta ₁X₁+\beta ₂X₂+\beta ₃X₃+\ldots +\beta_{3}X_{n})$$

Where,

- *Y*: Ordinal outcome (mobility limitation)
- *αⱼ*: Intercepts (thresholds between levels)
- *X₁…X_n_*: Predictors (e.g., income, pain, distance)
- *β₁…β_n_*: Regression coefficients

**Model 6: Chi-Square Test (Coping Strategies)**: To analyze perceived effectiveness of winter coping strategies among RA women.

**Equation:**


$$\chi^{2}=\sum [{\left(O_{i}-E_{i}\right)}^{2}/E_{i}]$$

Where,

- Same as Model 4

**Model 7: Structural Equation Modeling (SEM)**: To assess direct and indirect effects between socioeconomic, climate, and psychological variables on RA outcomes.

**Equation:**


βX+ζ.

Where,

- *Y*: Endogenous variables (e.g., pain, mobility, discontinuation)
- *X*: Exogenous variables (e.g., income, stress, season)
- *β*: Path coefficients (standardized estimates)
- *ζ*: Error terms

### 4.7 Ethical considerations

The study protocol was approved by the Research Ethics Committee of the University of Malakand (Ref No: UoM/REC/SOC/2025/116). All participants provided informed consent, with verbal consent audio-recorded for those with limited literacy following the WHO guidelines.

## 5 Results

Table 3 investigates the relationship between household income and RA pain intensity among women in District Dir Lower, directly addressing Objective 1 of the study: *“To examine how household income levels influence RA pain severity among women.”* Using the Kruskal–Wallis H Test, a non-parametric method appropriate for ordinal outcomes like pain ratings, the results reveal a statistically significant difference in pain severity across income levels (H(2) = 38.73, *p* < 0.001).

**Table 3:** Relationship between household income and RA pain intensity [Kruskal–Wallis H Test (Objective 1), *N* = 300].

Income level (PKR)	N	Median RA pain score (1–5)	Mean rank
< 15,000	110	4	187.2
15,000–30,000	130	3	141.6
> 30,000	60	2	93.3

Kruskal–Wallis H(2) = 38.73, *p* < 0.001, Lower-income groups reported significantly higher pain intensity.

The first income group, earning less than PKR 15,000, shows a median pain score of 4 (on a scale from 1 = no pain to 5 = severe pain) and a mean rank of 187.2. This clearly indicates higher RA pain intensity among the lowest-income participants. This empirical result supports the study’s premise that poverty intensifies the physical burden of RA, likely due to limited affordability of regular medical care, nutritious diets, pain management resources, and appropriate winter protection. In the middle-income group (PKR 15,000–30,000), the median pain score drops to 3 with a mean rank of 141.6, showing a moderate level of RA pain. This result confirms the gradient relationship between income and pain—as household financial capacity improves, the experience of pain diminishes. This supports the hypothesis that economic stability plays a buffering role against chronic disease severity.

Finally, women in the high-income group (earning above PKR 30,000) report the lowest median pain score of 2 and a mean rank of 93.3. This strongly aligns with the study’s assumption that higher-income women face significantly lower pain intensity, probably due to better healthcare access, routine consultations with rheumatologists, and affordability of indoor heating and medication—factors explored in other objectives as well.

Table 4 presents the comparison of RA pain severity across two seasons—summer and winter—using the Wilcoxon Signed-Rank Test, which is suitable for comparing paired ordinal data from the same participants. This analysis directly supports Objective 2 of the study: *“To assess the impact of seasonal changes on the intensity of RA pain among women in District Dir Lower.”*

**Table 4:** Comparison of RA pain severity by season [Wilcoxon signed-rank test (Objective 2), *N* = 300].

Season	Median pain score
Summer	2
Winter	4

Z = −12.24, *p* < 0.001, Effect size (r) = 0.52, Pain scores were significantly higher in winter than in summer.

The results show a clear seasonal disparity in reported pain scores. During summer, the median pain score was 2, which indicates a relatively mild level of pain on the 1–5 ordinal scale (1 = no pain, 5 = severe pain). In contrast, during winter, the median pain score sharply increases to 4, reflecting a substantially more severe pain experience among the same respondents.

Statistical analysis using the Wilcoxon Signed-Rank Test produced a Z-value of −12.24, which is highly significant (*p* < 0.001), confirming that this increase in pain severity from summer to winter is not due to random chance. Furthermore, the effect size (r) = 0.52 is considered large, suggesting a strong practical and clinical significance of seasonal variation in pain experiences.

This finding directly reinforces the study’s climate-health hypothesis that cold temperatures and harsh winter conditions exacerbate RA symptoms, particularly among women in rural areas like District Dir Lower, where access to heating, joint-protective clothing, and consistent medical care may be limited.

Table 5 investigates the relationship between spousal employment status and the stress levels of women diagnosed with Rheumatoid Arthritis (RA) using the Mann–Whitney U Test. This test is appropriate for comparing stress scores between two independent groups where the outcome variable (stress level) is measured on an ordinal scale (1–5). This analysis directly addresses Objective 4 of the study: *“To examine the psychological impacts—specifically stress levels—associated with socioeconomic variables among women living with RA in District Dir Lower.”*

**Table 5:** Stress levels among women by husband’s employment status [Mann–Whitney U Test (Objective 4), *N* = 300].

Husband’s employment status	N	Median stress score (1–5)	Mean rank
Employed	190	2	120.8
Unemployed	110	4	198.1

Mann–Whitney U = 5690.5, *p* < 0.001, Spousal unemployment is associated with significantly higher stress levels.

The results show that women whose husbands were unemployed reported substantially higher stress levels, as reflected by a median stress score of 4, compared to a median of 2 among those whose husbands were employed. This two-point difference on a five-point scale is clinically meaningful, indicating a significant psychological burden linked to economic insecurity.

The mean rank further supports this disparity: women with unemployed spouses had a mean rank of 198.1, while those with employed spouses had a considerably lower mean rank of 120.8. Since higher mean ranks in the Mann–Whitney U Test correspond to higher stress levels, this confirms that women with unemployed husbands experience markedly elevated stress.

The statistical result of the Mann–Whitney U Test is U = 5690.5, with a *p*-value < 0.001, indicating that the difference in stress levels between the two groups is statistically significant and unlikely to be due to chance.

These findings provide strong evidence that spousal unemployment—used here as a proxy for economic vulnerability—significantly contributes to elevated stress among women with RA. This aligns with the study’s broader theoretical framework, which emphasizes how socioeconomic strain (like lack of household income due to spousal unemployment) exacerbates psychological stress, potentially worsening RA outcomes.

Table 6 explores the association between distance to a rheumatologist and the likelihood of treatment discontinuation among women with Rheumatoid Arthritis (RA), analyzed using the Chi-square test of independence. This analysis addresses Objective 3 of the study: *“To assess how access barriers, particularly distance to specialized care, influence treatment continuity for women living with RA in District Dir Lower.”*

**Table 6:** Association between distance to rheumatologist and treatment discontinuation [Chi-square test (Objective 3), *N* = 300].

Distance to rheumatologist	Discontinued treatment (n)	Continued treatment (n)
< 10 km	18	42
10–50 km	53	67
> 50 km	74	46

Chi-square (2, *N* = 300) = 28.49, *p* < 0.001, Greater distance significantly increases likelihood of treatment discontinuation.

Among participants living within 10 km of a rheumatologist, only 18 women discontinued treatment, while 42 continued, suggesting relatively better access and care continuity in this group. For those living 10–50 km away, 53 women discontinued treatment, compared to 67 who continued, indicating a moderate barrier effect. However, the most striking results appear for participants living more than 50 km away: 74 women discontinued treatment, while only 46 continued. This sharp increase in discontinuation reflects the severe logistical and economic challenges of accessing distant care.

The Chi-square value of 28.49 with 2 degrees of freedom, and a *p*-value < 0.001, indicates that the relationship between distance and treatment discontinuation is statistically significant. In simpler terms, the further a woman lives from a rheumatologist, the more likely she is to discontinue treatment, and this relationship is not due to chance.

This finding aligns directly with the study’s broader hypothesis that healthcare access barriers—such as physical distance—serve as a major determinant of health outcomes in marginalized, rural populations. Women in remote areas, often lacking private transport or financial means for regular travel, face systemic obstacles that force them to delay, interrupt, or abandon medical care.

The results of the ordinal logistic regression presented in Table 7 reveal key predictors of winter mobility limitation among women suffering from Rheumatoid Arthritis (RA) in District Dir Lower. The analysis demonstrates that low household income significantly increases the odds of severe mobility impairment in winter, with women earning less than PKR 15,000 being 4.22 times more likely to report higher levels of disability (B = 1.44, *p* < 0.001). Similarly, spousal unemployment was found to be a strong predictor, where women whose husbands were unemployed had 3.22 times greater odds of mobility limitations (B = 1.17, *p* < 0.001). Most notably, the intensity of RA pain during winter emerged as the most powerful predictor; with every unit increase in pain severity, the odds of experiencing mobility difficulty increased by nearly seven times (Odds Ratio = 6.89, B = 1.93, *p* < 0.001). Additionally, geographic barriers played a critical role—those living more than 50 km away from a rheumatologist were 2.41 times more likely to discontinue treatment and report winter mobility challenges (B = 0.88, *p* = 0.001). The model demonstrated a good fit, with Nagelkerke R^2^ = 0.43, suggesting that these variables collectively explain 43% of the variance in winter mobility limitations. These findings align with the broader aims of the study, highlighting how socioeconomic hardship, gendered stress, environmental exposure, and healthcare inaccessibility intersect to intensify the impact of RA. The results affirm that winter pain and structural inequalities are not isolated challenges but mutually reinforcing burdens that demand integrated medical and social interventions.

**Table 7:** Predictors of winter mobility limitation [Ordinal logistic regression (Objective 1, 2, 4), *N* = 300].

Predictor variable	B (β coefficient)	SE	Wald χ^2^	*p*-value	Odds ratio (Exp(B))
Low income (<15,000 PKR)	1.44	0.32	20.25	<0.001	4.22
Spousal unemployment	1.17	0.28	17.50	<0.001	3.22
Pain severity in winter (1–5)	1.93	0.41	22.74	<0.001	6.89
Distance to rheumatologist (>50 km)	0.88	0.26	11.47	0.001	2.41

Model Fit: Nagelkerke R^2^ = 0.43, Winter pain severity and socioeconomic hardship significantly predict mobility disability.

Table 8 presents the findings from a Chi-square Test of Independence examining the perceived effectiveness of various coping strategies employed by women with Rheumatoid Arthritis (RA) during winter in District Dir Lower. The results show a statistically significant association between the type of coping strategy and its perceived effectiveness, χ^2^(3, *N* = 300) = 29.84, *p* < 0.001. Among the respondents, indoor heating emerged as the most effective strategy, with 74 out of 95 women reporting it as beneficial in managing winter-related RA symptoms. This aligns with the environmental focus of the study, confirming that access to climate-control solutions can mitigate winter pain and enhance daily functioning for women in low-resource settings.

**Table 8:** Coping strategies in winter among women with RA [Chi-square test of independence (Objective 5), *N* = 300].

Coping strategy	Effective (n)	Not effective (n)	Total
Indoor heating	74	21	95
Traditional remedies	56	49	105
Physical inactivity/rest	31	39	70
Continued outdoor work	12	18	30

Chi-square (3, *N* = 300) = 29.84, *p* < 0.001, Indoor heating was perceived as the most effective coping strategy.

Traditional remedies, such as herbal applications and homemade ointments, were used by 105 women; however, only 56 found them effective while 49 did not, indicating mixed results and suggesting a cultural reliance on but variable trust in indigenous health practices. This finding reflects the intersection of gendered health behavior and limited access to formal care, as discussed in the study’s theoretical framework.

Physical inactivity or rest was used by 70 women, with only 31 rating it effective and 39 reporting it ineffective. While rest may offer temporary relief, prolonged inactivity may exacerbate stiffness and contribute to reduced mobility, echoing the study’s findings regarding winter-induced disability.

Lastly, continued outdoor work—often driven by financial necessity—was reported by 30 women, with only 12 finding it effective and 18 not. This highlights the role of socioeconomic pressure in shaping coping behaviors that may inadvertently worsen RA symptoms.

Table 9 presents the results of the Structural Equation Modeling (SEM) conducted to test the integrated pathways among socioeconomic, environmental, psychological, and health-related determinants of Rheumatoid Arthritis (RA) outcomes in District Dir Lower. The SEM model shows excellent fit with the data, as indicated by key model fit indices: the Comparative Fit Index (CFI) = 0.94, the Tucker–Lewis Index (TLI) = 0.91, Root Mean Square Error of Approximation (RMSEA) = 0.042, and Chi-square/df = 1.92—all within acceptable thresholds, confirming that the theoretical framework aligns well with the observed data.

**Table 9:** Structural equation modeling results [SEM model fit (all objectives), *N* = 300].

Path	Standardized estimate (β)	*p*-value
Income → Pain severity	−0.41	<0.001
Spousal unemployment → Stress	0.36	<0.001
Pain severity → Mobility limitation	0.54	<0.001
Winter season → Pain severity	0.47	<0.001
Stress → Treatment discontinuation	0.29	0.002
Distance → Treatment discontinuation	0.33	<0.001
Model fit indices		
Statistics	Value	
CFI	0.94	
RMSEA	0.042	
TLI	0.91	
χ^2^/df	1.92	

The SEM shows robust direct and indirect effects between socioeconomic status, climate sensitivity, psychological stress, and RA disability outcomes.

The path coefficients illustrate statistically significant relationships between key variables. The path from household income to pain severity yielded a standardized estimate (*β*) = −0.41, *p* < 0.001, showing that lower income is strongly associated with higher RA pain intensity. This supports Objective 1 of the study, emphasizing how economic hardship exacerbates physical symptoms of chronic illness.

The impact of spousal unemployment on women’s stress is also significant (*β* = 0.36, *p* < 0.001), reflecting Objective 4. This confirms the gendered burden of economic instability and validates prior findings from Table 5, where unemployed husbands were linked to higher median stress levels in their wives.

Furthermore, pain severity was positively associated with winter mobility limitation (*β* = 0.54, *p* < 0.001), aligning with Objectives 1 and 2. It supports earlier regression findings that winter pain significantly impedes physical function, particularly among socioeconomically vulnerable women.

The winter season itself directly influenced pain severity (*β* = 0.47, *p* < 0.001), echoing Wilcoxon test results from Table 4 and reinforcing the environmental sensitivity component of the study. Cold weather appears to substantially intensify pain, making this a key seasonal determinant of RA disability.

Psychological stress also predicted treatment discontinuation (*β* = 0.29, *p* = 0.002), demonstrating the indirect ways in which social pressures affect health adherence. Similarly, distance to the rheumatologist showed a significant positive path to treatment discontinuation (*β* = 0.33, *p* < 0.001), affirming earlier Chi-square results and underscoring how infrastructural challenges contribute to poor care continuity.

## 6 Discussion

The results of this study highlight a complex and multidimensional relationship between socioeconomic status, seasonal variation, psychological distress, healthcare access, and functional disability among women with Rheumatoid Arthritis (RA) in rural Pakistan. The findings revealed that women from lower-income households experienced more severe RA pain, which aligns with previous global studies demonstrating how poverty exacerbates the symptoms of chronic illness due to limited access to healthcare, nutritious food, and disease management resources (34, 35). Economic hardship constrains both preventive care and treatment adherence, a challenge compounded in rural regions with fragile health infrastructure.

Seasonal effects were also pronounced, with winter months associated with significantly greater pain intensity and functional limitation. This echoes research from temperate and subtropical climates where colder temperatures have been linked to worsened musculoskeletal symptoms (36). Studies conducted in Iran and northern India have similarly shown that RA patients report increased stiffness, pain, and difficulty in mobility during winter, especially among those with inadequate heating or protective clothing (37, 38). These findings support the climate-health nexus highlighted in the present study, confirming that environmental factors play a vital role in symptom severity and quality of life for RA patients.

Psychosocial stress was also significantly higher among women whose spouses were unemployed. This supports earlier literature indicating that economic dependence and household financial insecurity serve as chronic stressors for women managing long-term illness (39, 48, 49). Psychological strain has been shown to interfere with self-care, contribute to treatment fatigue, and ultimately worsen physical outcomes. In South Asian contexts where women’s healthcare access is often mediated by male employment and decision-making, spousal unemployment functions as both an economic and emotional stressor (40).

In terms of healthcare access, women who lived farther from specialized rheumatologic services were substantially more likely to discontinue treatment. This finding corroborates previous studies in similar low-resource settings which identified physical distance and lack of transportation as critical barriers to continuous care (41, 42). These structural inequalities hinder early diagnosis, routine follow-ups, and disease monitoring factors essential for effective RA management.

The regression analysis revealed that winter pain severity, low income, spousal unemployment, and distance to care were all significant predictors of reduced mobility during winter. These findings are consistent with the bio-psychosocial model of chronic illness, which suggests that disability is not solely a function of physical symptoms but is also shaped by social and environmental stressors (43). In rural settings, where women are often primary caregivers and may lack autonomy over health decisions, such barriers can have especially pronounced effects on disease progression and mobility.

Coping strategies varied in effectiveness. While indoor heating was perceived as the most effective measure, reliance on traditional remedies and physical inactivity had more mixed outcomes. This finding parallels studies in Bangladesh and Nepal that reported limited efficacy of home-based or cultural remedies in managing inflammatory arthritis, especially in the absence of medical intervention (44, 45). The use of continued outdoor work despite pain was often driven by economic necessity, reflecting how gendered labor roles and poverty may force women to compromise their health.

The Structural Equation Modeling confirmed the interplay between all these variables, validating the study’s conceptual framework. Notably, it demonstrated both direct and indirect effects such as how stress mediated the relationship between unemployment and treatment discontinuation highlighting the multifactorial nature of RA outcomes. Similar integrated models have been employed in global health research, particularly in the study of chronic illness in low-income countries (46).

What distinguishes this study from prior work is its focused lens on women in a rural, conflict-affected district of Pakistan—a context where climate, poverty, gender inequality, and health infrastructure converge in unique ways. The findings demonstrates that women with RA in this setting face a multiple jeopardy (47), where their health outcomes are shaped not by a single factor, but by the synergistic interaction of their gender, economic marginalization, environmental vulnerability, and geographic isolation. While many studies (e.g., 50–53) have examined individual predictors of RA severity, this research integrate these environmental, social, and psychological dimensions into a unified analytical model. Furthermore, it provides localized evidence from a region underrepresented in global rheumatology literature, offering insights with both clinical and policy relevance for health systems in similar underserved settings.

## 7 Conclusion

Based on the comprehensive analysis of the data, the study concludes that RA outcomes among women in District Dir Lower are significantly shaped by a complex interplay of socioeconomic disadvantage, environmental stressors, psychological burden, and healthcare access barriers. The findings confirm that lower household income is strongly associated with heightened pain severity and increased functional limitations, reinforcing the argument that poverty intensifies chronic illness through restricted access to healthcare, nutrition, and winter coping resources. Seasonal variation also emerged as a critical factor, with winter conditions substantially exacerbating RA pain and disability, particularly among those lacking adequate heating or protective infrastructure.

Spousal unemployment was found to contribute meaningfully to psychological stress, highlighting the gendered dimensions of economic dependency and emotional strain. This stress, in turn, was associated with a greater likelihood of treatment discontinuation, underscoring how psychosocial pressures disrupt long-term disease management. Furthermore, geographic distance to specialist care was a consistent barrier to treatment continuity and mobility, confirming that structural and infrastructural inequities deeply affect healthcare utilization in rural settings.

Coping strategies employed by women during winter varied in effectiveness, with indoor heating emerging as the most beneficial, while reliance on traditional remedies or continued outdoor labor often proved less effective or even detrimental. Finally, the structural equation model validated the integrated framework of the study, demonstrating that these factors do not operate in isolation but interact dynamically to influence disease outcomes.

## 8 Policy implications

The findings of this study underscore the urgent need for integrated, gender-sensitive health policies that address the socioeconomic, environmental, and healthcare access determinants of RA among women in rural Pakistan. Policymakers must prioritize the decentralization of rheumatology services by establishing satellite clinics or mobile health units in underserved districts like Dir Lower, thereby reducing the burden of long-distance travel that significantly contributes to treatment discontinuation. Social protection programs should be expanded to support low-income and female-headed households affected by chronic illness, ensuring access to subsidized medications, heating resources during winter, and nutritional support. In light of the strong association between spousal unemployment and women’s psychological stress, employment schemes and conditional cash transfers targeting vulnerable families can indirectly alleviate health-related burdens by improving household stability. Moreover, public health campaigns and community-based interventions should raise awareness about the dangers of delaying treatment and the limited efficacy of traditional remedies, while promoting cost-effective and scientifically validated coping strategies such as thermal care and physical therapy. Climate-sensitive health planning—such as seasonal resource distribution and localized weather-based pain management alerts—can further support disease mitigation during winter months. Lastly, cross-sector collaboration between the health, social welfare, and women’s development departments is essential to design holistic interventions that recognize RA not merely as a clinical issue but as a condition shaped by poverty, gender, geography, and climate.

## 9 Limitations and future directions

This study, while comprehensive in its quantitative assessment of the socioeconomic, environmental, and gendered determinants of RA in District Dir Lower, carries certain limitations that future research should address. A primary limitation is the focus on patient-reported outcomes (e.g., self-reported pain, mobility limitation, and stress) without the inclusion of objective clinical metrics such as serological status (RF/CCP), Disease Activity Score 28 (DAS28), or radiographic findings. While this approach effectively captures the patients’ lived experience, it limits the ability to correlate the identified socio-structural factors with standardized biomedical measures of diseases activity and severity. The reliance solely on cross-sectional data restricts the ability to establish causality between variables such as income, seasonal change, and RA outcomes—future studies may benefit from longitudinal designs to track disease progression and response to interventions over time. Future research should integrate these clinical measures with socio-structural model presented here to build a more holistic, bio-psycho-social understanding of RA in this context. Moreover, the scope was geographically limited to one district in Khyber Pakhtunkhwa, potentially limiting generalizability to other socio-cultural or ecological zones across Pakistan. Furthermore, while the study was designed with a primary focus on women’s experiences, the inclusion of a very small number of male participants (*n* = 24) prevents any robust statistical gender-based comparisons and limits the exploration of male patients’ perspectives. Finally, the study did not explore the role of health literacy, social support networks, or digital health access, which may moderate treatment outcomes and coping behaviors. Future research should incorporate mixed-methods approaches to capture the subjective experiences of women living with RA and explore how structural inequalities and health system gaps intersect with individual resilience. Specifically, future studies should intentionally recruit a balanced sample of men and women to enable a comprehensive, comparative analysis of gender disparities in RA burden and management. Addressing these gaps will deepen understanding and contribute to more tailored, equity-focused interventions for chronic disease management in marginalized settings.

## Data Availability

The original contributions presented in the study are included in the article/supplementary material, further inquiries can be directed to the corresponding author.
